# The effectiveness of harvest for limiting wildlife disease: Insights from 20 years of chronic wasting disease in Wyoming

**DOI:** 10.1002/eap.3089

**Published:** 2025-01-21

**Authors:** Wynne E. Moss, Justin Binfet, L. Embere Hall, Samantha E. Allen, William H. Edwards, Jessica E. Jennings‐Gaines, Paul C. Cross

**Affiliations:** ^1^ U.S. Geological Survey Northern Rocky Mountain Science Center Bozeman Montana USA; ^2^ Wyoming Game and Fish Department Cheyenne Wyoming USA

**Keywords:** causal inference, chronic wasting disease (CWD), epidemiology, harvest, hunting, mule deer, prion disease, wildlife disease, wildlife management

## Abstract

Effective, practical options for managing disease in wildlife populations are limited, especially after diseases become established. Removal strategies (e.g., hunting or culling) are used to control wildlife diseases across a wide range of systems, despite conflicting evidence of their effectiveness. This is especially true for chronic wasting disease (CWD), an untreatable, fatal prion disease threatening cervid populations across multiple countries, for which recreational harvest has been suggested as an important disease control strategy. Using observational data to evaluate whether harvest effectively limits CWD prevalence has been difficult because statistical relationships between harvest and disease prevalence can arise from a causal effect of harvest (i.e., harvest's impacts on prevalence via changes in transmission or demographic structure) or from a number of alternative mechanisms. For instance, correlations between harvest and disease prevalence can also be driven by disease's impacts on population size and harvest (i.e., reverse causality) or from confounding variables (e.g., habitat or geographic location) that impact both harvest rates and disease prevalence. We analyzed two decades of surveillance data (2000–2021) from 10 mule deer herds in Wyoming, using statistical approaches informed by causal inference theory, to test for the effects of harvest on CWD prevalence. Herds with consistently high harvest pressure across 20 years had significantly lower prevalence. Our models predicted that harvesting 40% of adult males per year across 20 years would maintain prevalence below 5% on average, whereas if only 20% of males were harvested in each year, prevalence would increase to >30% by year 20. Moreover, shifting the relative harvest pressure within a herd over a shorter period (3 years) reduced subsequent prevalence, albeit to a smaller degree. Although high harvest is unlikely to completely eradicate CWD, our analysis suggests that maintaining hunting pressure on adult males is an important tactic for slowing CWD epidemics within mule deer herds. Our study also provides guidance for future analyses of longitudinal surveillance data, including the importance of demographic data and appropriate time lags.

## INTRODUCTION

Managing the spread and impacts of wildlife disease is a high priority not only for wildlife conservation but also for protecting human and livestock health, maintaining important ecological services, and sustaining recreational and economic opportunities (Conover et al., 1995; Daszak et al., 2000; Miller et al., 2013). However, wildlife disease management is challenged by a scarcity of effective, practical disease control strategies for free‐ranging populations, and, in many cases, uncertainty about pathogen biology and disease dynamics (Henke et al., 2007; Joseph et al., 2013). Early intervention strategies that limit disease introduction into naïve populations (e.g., restrictions on wildlife trade) are generally considered the most cost‐effective and feasible disease management approaches (Henke et al., 2007; Voyles et al., 2015). Yet, identifying approaches for controlling the endemic stages of diseases is also essential as global change furthers the emergence and spread of wildlife diseases worldwide, and because containment or eradication efforts often fail (Fenichel et al., 2010; Langwig et al., 2015; Tompkins et al., 2015).

After infectious disease has entered a population and eradication is unlikely, management focus often shifts to limiting transmission, reducing prevalence, or mitigating population impacts (Langwig et al., 2015). In these stages, removal‐based strategies that target focal wildlife hosts or reservoir species may be used and have been most commonly applied to diseases with high spillover potential like bovine tuberculosis, rabies, and brucellosis (Carter et al., 2009; Gortazar et al., 2015; Lambert et al., 2021; Miguel et al., 2020; Prentice et al., 2019). Removal strategies can be selective, seeking to target and eliminate infected individuals to control risk to susceptible populations or individuals. For instance, test‐and‐cull approaches have been used in African buffalo to identify and lethally remove individuals infected with bovine tuberculosis, effectively reducing transmission within buffalo populations and spillover to livestock (le Roex et al., 2016). Nonselective approaches, including density reductions through population‐level culling programs or recreational harvest (Carter et al., 2009), are also used to decrease density‐dependent transmission, although they can also work by shifting age–sex structure or shortening the infectious period (Potapov et al., 2012). While removal‐based strategies have the potential to reduce disease prevalence, they are far from universally effective, and in some cases, lead to unintended consequences. For example, density reductions may fail to reduce frequency‐dependent or highly heterogeneous disease transmission (Bolzoni et al., 2007; Lachish et al., 2010; Lloyd‐Smith et al., 2005), can require undesirable levels of population reduction (Bozzuto et al., 2020), or may trigger unexpected changes in host population structure or behavior (Donnelly et al., 2006; Prentice et al., 2019; Viana et al., 2023). At the same time, removal efforts are often controversial and difficult to implement (Holsman et al., 2010; White & Whiting, 2000). Management agencies risk eroding public support or, at worst, exacerbating the effects of disease‐driven declines if these programs do not work. Evaluations of removal‐based strategies based on empirical data are greatly needed to identify situations in which they can be confidently deployed.

**FIGURE 1:**
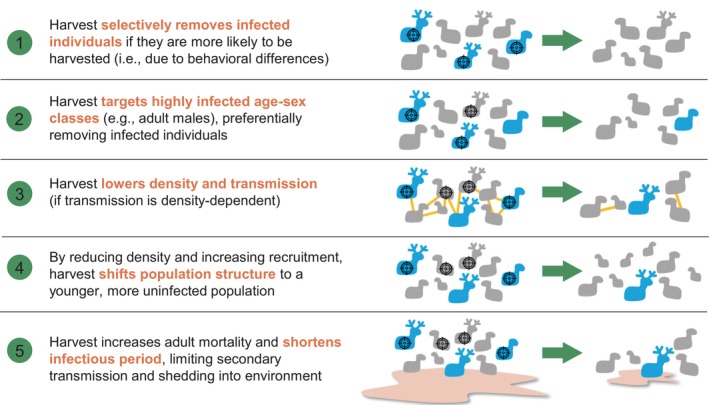
Potential mechanisms linking harvest to reductions in disease prevalence, which are not mutually exclusive. Infected individuals are depicted in blue. It is not yet understood which of these mechanisms are involved in harvest's effects on chronic wasting disease, although several studies have discussed the potential for these mechanisms to operate: (1) Conner et al. (2000), Edmunds et al. (2016); (2) Jennelle et al. (2014), Potapov et al. (2016), Samuel (2023); (3) Schauber and Woolf (2003), Habib et al. (2011), Mysterud and Edmunds (2019); (4) Potapov et al. (2012). Importantly, some mechanisms directly slow transmission and reduce the force of infection, whereas others rely upon shifting population structure. The optimal harvest strategy (i.e., which individuals or age–sex classes are targeted) also varies by mechanism, as depicted by the cross hairs.

The difficulty of managing wildlife disease is exemplified by chronic wasting disease (CWD), an infectious, neurodegenerative prion disease that has had considerable ecological, cultural, and economic impacts since it was first recognized in the 1960s (Chiavacci, 2022; DeVivo et al., 2017; Edmunds et al., 2016; Heberlein, 2004; Schroeder et al., 2022; Vaske et al., 2004). CWD is slow spreading, 100% fatal, and no effective treatments have been developed to date (Williams et al., 2002). Despite significant efforts to contain its spread, CWD has now expanded to six countries, 35 US states, and four Canadian provinces (Richards, 2024). CWD affects cervids, including important game species, and thus, recreational harvest could be a plausible strategy for managing CWD. The belief that harvest could limit CWD spread has led to its adoption as a control strategy in numerous management agencies across the United States and Canada (Brandell et al., 2022; Thompson et al., 2023). Yet, despite several potential mechanisms linking harvest and CWD prevalence (Figure 1), uncertainty remains about whether harvest strategies are actually effective in real populations. Simulation studies suggest the effectiveness of harvest is sensitive to assumptions about density dependence, transmission dynamics, harvest strategy, and contribution of the environmental pool, and several of these assumptions have not been empirically tested (Almberg et al., 2011; Jennelle et al., 2014; Potapov et al., 2016; Rogers et al., 2022; Uehlinger et al., 2016; Wasserberg et al., 2009).

**FIGURE 2:**
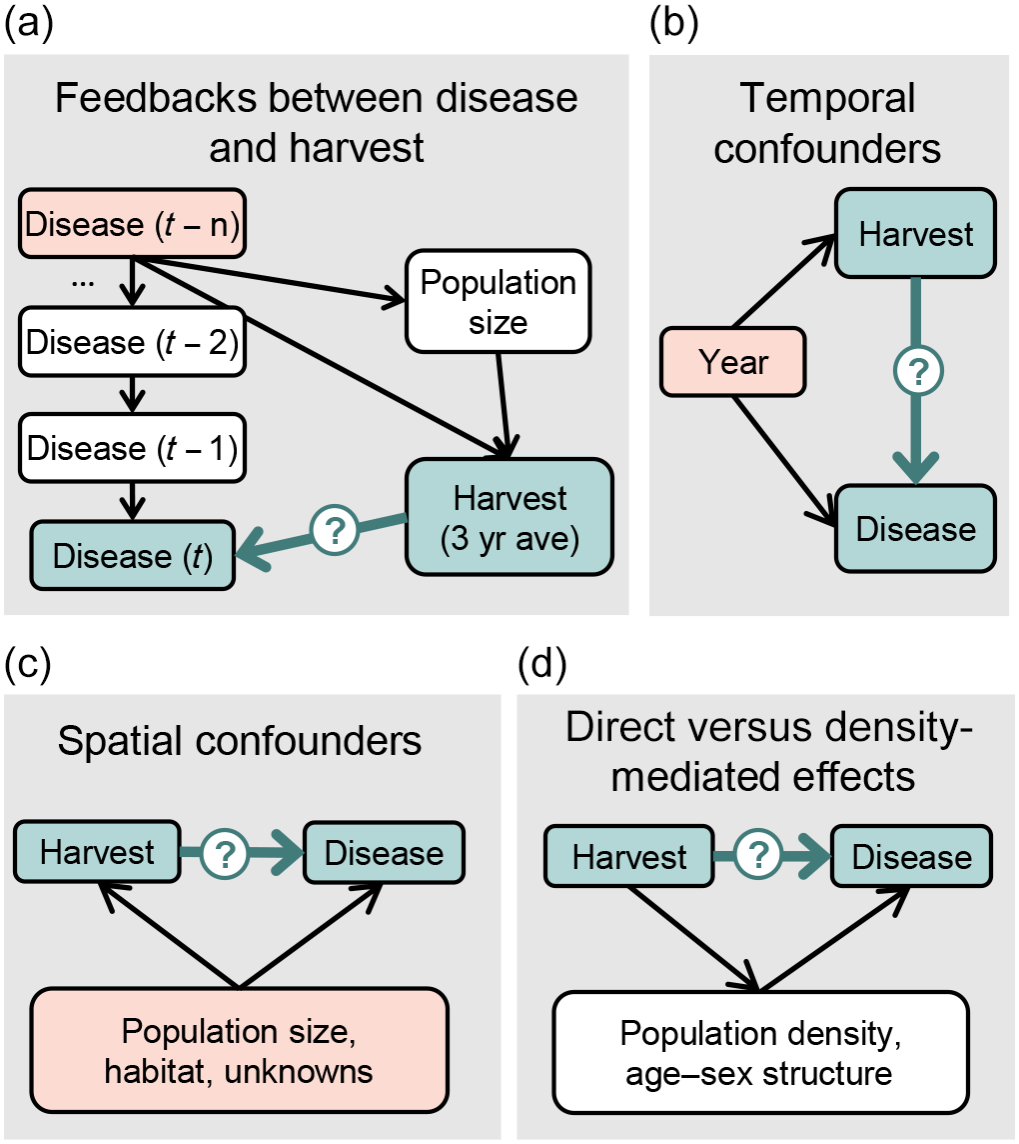
Potential issues that could affect the estimation of harvest's effect on infectious disease. The relationship of interest (in blue) is the effect of harvest on chronic wasting disease (CWD) prevalence. Potential confounding variables are in pink. (a) Previous years' prevalences may represent a confounder, affecting both harvest and current prevalence, because disease can impact population size and subsequent harvest, and because agencies may directly respond to disease by adjusting harvest quotas. (b) Disease prevalence and harvest may have independent temporal trends, creating a spurious statistical relationship between them. (c) Disease prevalence and harvest may be confounded by factors like population size and habitat (or unknown confounders). (d) Harvest may directly affect disease prevalence (e.g., by removing infected individuals) or may affect prevalence by altering demographic variables like age–sex structure or density. While not confounding variables, the inclusion of these mediating variables affects the estimation of harvest's effects.

Observational studies estimating the impacts of recreational harvest on CWD are still scarce (Uehlinger et al., 2016), with a few exceptions. Higher rates of male harvest were associated with reduced prevalence in male mule deer across a range of jurisdictions in the western United States and Canada (Conner et al., 2021). In Colorado, declines in licenses were associated with increased prevalence in mule deer (Miller et al., 2020). Both studies related hunting on one‐ to two‐year lags to prevalence in subsequent years using linear regression models. Inferring a causal effect of harvest on disease prevalence using observational data is difficult for several reasons. Harvest pressure is not applied randomly to populations, and it may correlate with confounding variables like density or habitat, which may also impact disease dynamics. In many jurisdictions, the number of hunters or licenses have declined over the past few decades (Miller et al., 2020; U.S. Fish and Wildlife Service, 2023; U.S. Fish and Wildlife Service and U.S. Census Bureau, 2016), while CWD has simultaneously increased during invasion phases. Moreover, many agencies have directly responded to the threat of CWD by changing harvest management (Brandell et al., 2022; Thompson et al., 2023), making it difficult to isolate the effects of harvest on CWD versus the potential impact of CWD on harvest. All of these factors may create correlations between harvest and CWD that could be misinterpreted as a causal relationship (Figure 2). Careful consideration of how to statistically isolate the effects of hunting from other confounding variables and feedbacks is needed to provide robust evidence on the effectiveness of harvest‐based strategies. The field of causal inference provides a helpful framework for tackling several of these issues, including methods for identifying noncausal statistical associations between variables (Laubach et al., 2021; Shipley, 2016). Approaches from causal inference are increasingly being used in applied ecology contexts (e.g., Hone et al., 2023; Simler‐Williamson & Germino, 2022; West et al., 2022), where estimating causal (and not just correlational) relationships is needed to increase confidence in potential management interventions.

Herein, we analyze over two decades of CWD surveillance data from mule deer (*Odocoileus hemionus*) herds in Wyoming to estimate the effects of recreational harvest on CWD dynamics. We use models informed by causal inference theory to better isolate the effects of harvest and control for feedbacks and confounding variables. Our analysis broadly focused on three questions: (1) How does spatial variation in harvest pressure contribute to different temporal trends in CWD prevalence across populations? (2) How do changes in harvest within a population across relatively short timescales alter subsequent CWD prevalence? (3) Which metrics of harvest pressure best relate to CWD prevalence? Through a combination of carefully selected analyses, our study provides robust empirical evidence regarding how harvest affects a high‐priority wildlife disease.

## METHODS

### Study populations

**FIGURE 3:**
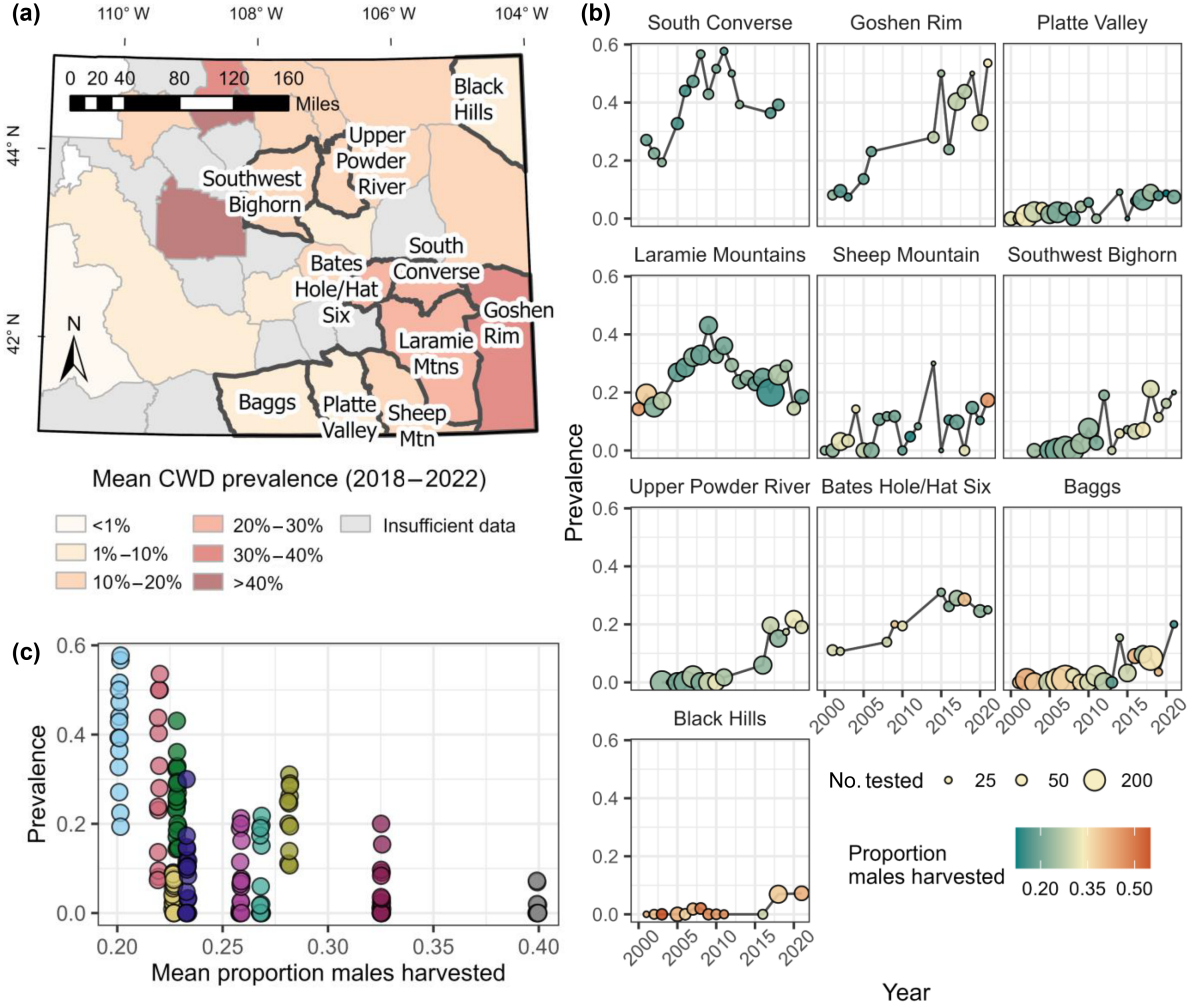
(a) Mule deer ( *Odocoileus hemionus* ) herds in Wyoming (outlined in black), for which we analyzed chronic wasting disease (CWD) prevalence from 2000 to 2021 (1 mile = 1.6 km). (b) Temporal trends in CWD prevalence for each herd, with herds arranged in order of lowest to highest average harvest pressure. We plot prevalence in adult males and show only those years with at least 10 adult males tested. (c) The relationship between prevalence and mean harvest pressure across mule deer herds, with each color depicting a different herd.

We analyzed data from 10 mule deer herds in Wyoming, which varied in prevalence and hunting effort, but for which CWD surveillance effort was relatively consistent (Appendix S1: Table S1). Herds were distributed across the central and eastern portion of the state (Figure 3a). In Wyoming, mule deer herds are defined as discrete populations having <10% interchange between adjacent populations. The total area occupied by each herd ranged from 2660 to 13,715 km^2^, whereas the potential winter range (see Appendix S1: Supplemental Methods: Covariates) of each herd ranged from 1888 to 13,676 km^2^ (Appendix S1: Table S1).

### Harvest management

Each year, harvest prescriptions for all big‐game herds in Wyoming are formulated and approved through a public‐input process, in an effort to provide hunting opportunity while managing populations and male ratios toward defined management objectives. Across the past several decades, mule deer population declines have been observed across Wyoming and have been partially attributed to CWD (DeVivo et al., 2017) as well as factors like energy development and climate (Sawyer et al., 2017). Population declines have resulted in increasingly conservative prescribed harvest over the past two decades, including the elimination of significant female harvest in most herds as well as substantially reduced license issuance and male harvest in many herds (Appendix S1: Figure S1). Some mule deer herds in Wyoming are managed under a limited quota system, in which the number of licenses available (and thus harvest pressure) can be strongly manipulated by managers. However, many of the herds in our study are managed under a general license structure (Appendix S1: Table S1), which allows unlimited resident licenses. Approaches used to modify harvest pressure in these herds include changes in the number of nonresident licenses, shifts in season length, and antler point restrictions.

CWD was first detected in Wyoming's free‐ranging mule deer in 1985 in the southeastern portion of the state and has since spread to 35 of 37 of the state's mule deer herds. In response to concerns, toward the end of our study period, the Wyoming Game and Fish Department (WGFD) revised its Chronic Wasting Disease Management Plan to incorporate more robust disease surveillance and adopt specific disease management strategies, like targeted hunter harvest (Wyoming Game and Fish Department, 2020). However, unlike some other US states that directly increased harvest quotas in response to CWD detection (Thompson et al., 2023), harvest‐based disease management strategies were not widely employed in Wyoming during the course of our study, outside of a few focal areas (Wyoming Game and Fish Department, 2020).

### CWD surveillance

Our analyses focused on patterns of CWD prevalence in adult male mule deer from 2000 to 2021. Annual prevalence in adult males was estimated using hunter‐harvested samples collected as part of WGFD's statewide surveillance program (Wyoming Game and Fish Department, 2020). Surveillance effort within each herd varied through time (Figure 3b); prior to 2019, efforts were concentrated on monitoring the leading edge and estimating prevalence within the core endemic area in southeastern Wyoming and later changed to a rotation‐based statewide surveillance program in which each herd receives heightened sampling effort roughly every 5 years (Wyoming Game and Fish Department, 2020). Hunter participation in the surveillance program was voluntary during our study period. In order to conduct a longitudinal analysis, we selected 10 herds for which there was a relatively consistent surveillance effort over the past two decades, consisting of at least 10 years where >20 adult males were tested. CWD is known to have been present since at least 2004 in all 10 of the herds.

Prior to 2003, infection status was determined using immunohistochemistry performed by the Wyoming State Veterinary Laboratory. After 2003, the majority of samples were tested using enzyme‐linked immunosorbent assays (ELISAs) of retropharyngeal lymph nodes or obexes at WGFD's Wildlife Health Laboratory (Hibler et al., 2003; Jennelle et al., 2009). In situations where new CWD areas were detected or ELISA results were conflicting, immunohistochemistry was performed to confirm ELISA results. We aggregated individual‐level testing results into an annual prevalence estimate for each herd (number of adult males testing positive/total number of adult males tested). We did not filter out years with low sample sizes; however, analyses were weighted by sample size such that years with poor sampling contributed less to model estimates (see *Autoregressive model*, below).

### Covariate data

Our analysis used annual estimates of harvest rates and demographic variables derived from WGFD's annual population surveys and big game reports. Demographic covariates used in our statistical models or in the computation of harvest pressure included buck:doe ratio, fawn:doe ratio, and total population size (for details, see Appendix S1: Supplemental Methods: Covariates). We also estimated density (in individuals per square kilometer) for each herd using the annual population size divided by the area of winter range (Appendix S1: Supplemental Methods: Covariates). This estimate represented densities during the time of year when individuals are most likely to be aggregated, which may be more relevant for disease transmission (Miller & Conner, 2005). However, we note that, in the absence of GPS‐collar data, the exact area used by each herd in each year is difficult to assess, and thus, the estimates derived herein should be interpreted as only a rough index of density.

To understand the impacts of harvest on CWD dynamics, we explored several different potential metrics of harvest pressure, which varied in their ease of data collection and degree of manager control. Harvest variables included the total number of adult males (yearling or older) harvested (*totalmale*), the proportion of adult males in the population that were harvested (*propmale*), the number of licenses sold (*licensesold*), and the number of active licenses per deer (*activelicenseperdeer*). Essentially, *propmale* and *activelicenseperdeer* are harvest metrics standardized by population size. For more information on how these variables were estimated, see Appendix S1: Supplemental Methods: Covariates.

We estimated each metric over several different time windows: a lag of 1 year (i.e., hunting pressure at time *t* − 1), a 3‐year running average (i.e., the mean hunting pressure from time *t* − 3 through *t* − 1), and a herd‐level average, across all 21 years of our study. Lags of 1–2 years have been used in previous studies; however, given the slow dynamics of CWD epidemics, we also considered longer time lags (Conner et al., 2021; Miller et al., 2020). Because we were interested in how temporal variation in harvest pressure *within* a herd affected CWD prevalence, we also calculated *relative* harvest pressure by subtracting the herd‐level average from the 3‐year lagged variables. In other words, the 3‐year relative hunting pressure was the hunting pressure in the past 3 years compared with the herd's average.

### Analytical approach

Our goal was to estimate the effects of recreational harvest on CWD prevalence in adult mule deer using longitudinal, cross‐sectional surveillance data. We combined several statistical techniques (Appendix S1: Table S2), each of which used generalized linear mixed models to address different potential issues for making causal claims about the effects of harvest (Appendix S1: Supplemental Methods: Data Analysis). Briefly, our statistical approach involved (1) using autoregressive models to estimate the effects of harvest on subsequent prevalence while accounting for potential feedbacks between CWD prevalence and hunting; (2) testing for reverse causality, or the effects of CWD on harvest rates; and (3) evaluating whether results were robust to model structure. Our approach also investigated which metrics of harvest pressure and temporal windows best predicted changes in prevalence to offer insight on implementation of harvest strategies and inform collection of relevant data. All models were fit using R (version 4.3.0) using the package glmmTMB (Brooks et al., 2017).

### Autoregressive model

First, we used a generalized linear mixed model with an autoregressive (AR1) error structure to estimate the effects of harvest pressure on CWD prevalence. We selected this model, rather than a linear model including the effect of year (e.g., Conner et al., 2021), for several reasons. First, temporal patterns in CWD prevalence are nonlinear, even on a log‐odds scale. An autoregressive model better captured the hump‐shaped pattern of CWD prevalence over time that was observed in some herds, which may become more common over time as CWD enters an endemic phase (Figure 3b). Second, CWD prevalence in prior years strongly predicts prevalence in the next year, in part due to CWD's long infectious period and capacity for environmental persistence. An autoregressive model therefore reflects the temporally autocorrelated nature of these kinds of data. Third, accounting for previous year's prevalence in some way was needed to control for potential feedbacks between CWD prevalence and hunting, which allowed us to better isolate the effects of harvest on prevalence, rather than vice versa (Figure 2; Appendix S1: Supplemental Methods: Data Analysis).

The autoregressive models predicted CWD prevalence in adult males in year *t* in herd *k* as a function of (1) *mean* harvest pressure in herd *k*; (2) the *relative* harvest pressure in herd *k* in the previous 3 years; (3) the herd, as a random intercept term; and (4) previous CWD prevalence (i.e., the AR1 error term). The relative harvest pressure across the previous 3 years was used to evaluate how temporal changes in harvest within a herd affected subsequent prevalence, whereas the mean harvest pressure was used to examine how long‐term differences in harvest strategies affect patterns of CWD growth across herds (Appendix S1: Supplemental Methods: Data Analysis). We fit four separate models, each substituting one of four potential harvest pressure metrics: *propmale*, *activelicenseperdeer*, *totalmale*, or *licensesold* (models a1–a4; Appendix S1: Table S2). Prevalence was modeled as a binomially distributed variable, using a logit‐link function. Observations were coded as number of positive individuals out of total tested (i.e., number of successes out of *n* trials), and therefore, data were weighted by the number of individuals tested. The model was fit in R using the package glmmTMB (Brooks et al., 2017).

Harvest could affect CWD prevalence directly (e.g., by preferentially removing infected individuals) but also potentially through altering density (and density‐dependent transmission) and demographic structure (Figure 1). To evaluate whether some of the effects of harvest operated through changes in density and demographic ratios, we conducted a type of mediator analysis, which is a technique that estimates indirect effects by adding or removing mediator variables to a linear model (Laubach et al., 2021). We fit a fifth model (model a5), using *propmale* as the harvest metric and adding two additional predictors: density in the previous year and buck:doe ratio in the previous year (Appendix S1: Table S2). This “full” model estimated the effect of harvest after accounting for density and buck:doe ratio, in other words, the *direct* effect of harvest independent of changes to demography. The model *without* the two demographic predictors (model a1) estimated the *total* effect of harvest through both direct and indirect effects. We compared the effect of harvest between the two models (a1 and a5). If the effect of harvest is reduced to zero when density and buck:doe ratio are included in the model, it suggests that all of the effects of harvest operate through changes to these demographic variables.

All models were fit in R (version 4.3.0), and the fit was assessed using the package DHARMa (Hartig, 2022). We tested for overly influential points using the function “influence_mixed” in the package glmmTMB (Brooks et al., 2017).

### Reverse causality

We used linear models to examine the potential for reverse causality, that is, to understand whether CWD prevalence appeared to influence subsequent harvest rates (Appendix S1: Supplemental Methods: Data Analysis). We fit three separate models (models r1–r3; Appendix S1: Table S2) where the response variable was one of three metrics of relative harvest pressure (*propmale*, *activelicenseperdeer*, or *licensesold*) for herd *k* in year *t*. Each metric of harvest pressure was modeled as a normally distributed variable. Predictor variables were (1) year, (2) previous CWD prevalence (averaged over the prior 3 years), and (3) herd, as a random intercept. We only included years in which the 3‐year running average of CWD prevalence was based on a sample size of 200 or greater.

### Robustness checks

Although we based our modeling approach on knowledge of potential relationships among key variables in this system (Figure 2), we acknowledge that other relationships are possible. A key component of inferring causal relationships is the observation of robustness in parameter estimates. That is, the estimated effect of a variable (e.g., harvest pressure) should remain relatively stable across multiple models (Addicott et al., 2022). To test this, we fit a series of 12 generalized linear mixed models varying in model structure and the inclusion of additional covariates (Appendix S1: Table S3). We evaluated whether the effect of harvest on prevalence (i.e., the parameter estimate) was similar across these differing model forms and whether certain measures of harvest (e.g., absolute vs. relative measures of harvest, one to 3‐year time lags) were more robust than others.

Lastly, we used simulations to evaluate whether parameter estimates were affected by uncertainty in covariates (Appendix S2). We focused on the covariate *propmale* as it is derived from a combination of estimates produced by population models, harvest surveys, and population counts, all of which entail their own measurement errors; thus, this covariate is likely associated with the highest degree of measurement error (Appendix S1: Supplemental Methods: Data Analysis). We estimated the total measurement error on *propmale* by combining uncertainty from each of these data streams, using estimates of uncertainty reported by WGFD. We then simulated datasets for which measurement error was added to *propmale* and fit model a1 (Appendix S1: Table S2) to each of those simulated datasets. We compared parameter estimates from 100 simulated datasets to the original dataset to evaluate robustness (Appendix S2).

## RESULTS

Between 2000 and 2021, a total of 14,796 adult males were tested, with 1681 (11%) testing positive. Herds varied widely in their epidemic trajectories, with some herds exhibiting annual prevalence of >40%, while others remained relatively lower (<20%) for the duration of the study period (Figure 3b). Harvest pressure varied strongly across herds (Appendix S1: Table S1), with the mean proportion of males harvested ranging from 0.20 to 0.40. The proportion of males harvested was correlated with another metric of harvest pressure, active licenses per deer (*r* = 0.69); therefore, we did not include both metrics in the same model and focused mostly on the proportion of males harvested. The number of males harvested generally decreased through time; however, because the total number of males within each herd simultaneously declined, the proportion of males did not exhibit strong trends over the study period (Appendix S1: Figure S1).

### Autoregressive model

Across herds, higher mean rate of male harvest over the 20‐year timespan was strongly associated with lower CWD prevalence (model a1: β_mean_propmale_ = −14.8 ± 6.9 (SE); *p* = 0.03). Modifying relative male harvest pressure within a herd over a 3‐year period had a lower magnitude, but still significantly negative effect (model a1: β_3yr_rel_propmale_ = −5.0 ± 2.1; *p* = 0.01). The estimate for β_3yr_rel_propmale_ was sensitive to a few influential points; after removing them, the effect was slightly smaller (β_3yr_rel_propmale_ = −4.2 ± 2.1; *p* = 0.05). The inclusion of observed demographic variables (density and buck: doe ratio) did not appreciably change the estimated effect of male harvest (model a5: β_3yr_rel_propmale_ = −4.95 ± 2.5; *p* = 0.05; β_mean_propmale_ = −15.2 ± 6.5; *p* = 0.02). This suggests that the effects of harvest did not operate solely through changing population structure or density (Figure 1). Neither density nor male‐to‐female ratio had a significant effect on prevalence (model a5: β_density_ = −0.11 ± 0.11; *p* = 0.32; β_buck:doe_ = 0.00 ± 0.01; *p* = 0.41). Although the model with demographic variables included did not have substantially different parameter estimates, it did appear to fit the data slightly better than the one without, as determined by model diagnostics (Appendix S1: Figure S2), and thus, we focus on the parameter estimates from this model (a5). Overall, the model fit the observed data well, capturing the nonlinear trajectories observed in many herds (Appendix S1: Figure S3).

**FIGURE 4:**
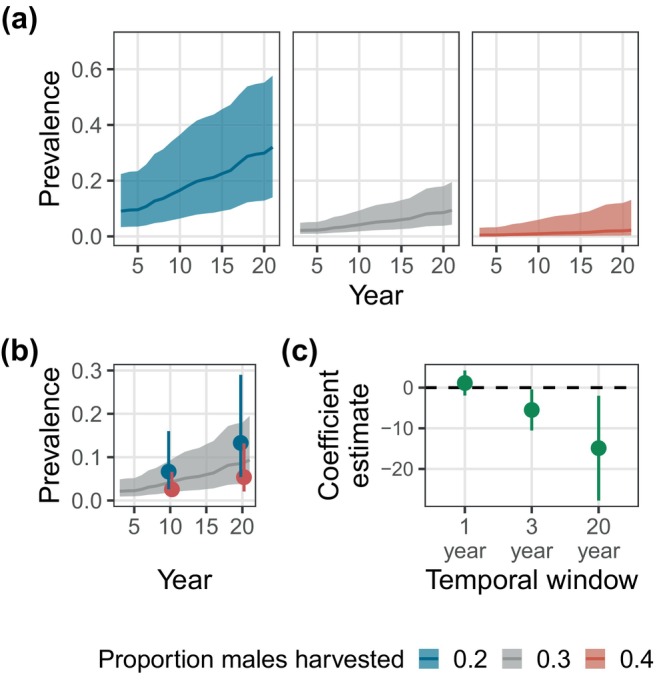
Predicted effects of long‐term vs. short‐term changes in harvest pressure on chronic wasting disease (CWD) prevalence. Harvest pressure is the proportion of males in the population that were harvested in a given year. (a) The predicted trajectories for CWD prevalence under three scenarios of mean harvest pressure. (b) The effect of a 3‐year increase or decrease in harvest pressure for a herd with a mean harvest pressure of 0.30. Points show the expected effect on prevalence for a change in harvest pressure applied at Year 10 or Year 20. Changes in the harvest pressure over 3 years affect prevalence, but to a lesser degree. (c) The effect size of harvest pressure when different temporal windows of harvest pressure were used. The previous year's harvest pressure had no significant effect on prevalence, whereas the harvest pressure across the past 3 years or past 20 years had significantly negative effects on prevalence. For each plot, the shading represents the 95% confidence interval for a single (average) herd. Predictions are derived from an autoregressive model (model a5; see Appendix S1: Table S2).

Using model predictions, a herd where 40% of males were harvested each year for two decades was predicted to have a prevalence of just 0.02 in year 20 (95% CI: 0.0–0.13), compared with a prevalence of 0.32 if only 20% of males were harvested on average (95% CI: 0.14–0.57; Figure 4a). Increasing the harvest pressure for just 3 years at the end of the study would lower prevalence to 0.13, compared with <0.05 if that level of harvest had been implemented for 20 years (Figure 4b). This was consistent with patterns in the raw data, in which differences in harvest pressure were strongly associated with differences in prevalence across herds, but patterns were less clear for changes in harvest pressure within a herd (Appendix S1: Figure S4). While increasing harvest for only a short period was less effective, our models do suggest that a sufficiently high increase in harvest applied over 3 years could reduce prevalence (instead of just slow its increase). However, for many herds, this level of harvest pressure was not achieved (Appendix S1: Figure S5).

Other metrics of harvest pressure, including the number of active licenses per deer, the number of licenses sold, and the number of males harvested, generally had no significant relationship with CWD prevalence (models a2–a4; Appendix S1: Table S4). Only the number of males harvested had a significant relationship with CWD prevalence (β_mean_totalmale_ = −2.01 ± 0.89; *p* = 0.02). However, we note that the effect of total male harvest was inconsistent across different model parameterizations (see *Robustness checks*).

### Reverse causality

We found no evidence of reverse causality, such that CWD prevalence in the prior 3 years did not appear to affect hunting pressure in subsequent years. This was true whether we tested the effects of CWD on the proportion of males harvested (model r1: β_3yr_CWD_ = 0.14 ± 0.09; *p* = 0.12), the number of active licenses per deer (r2: β_3yr_CWD_ = 0.01 ± 0.01; *p* = 0.12), or the number of licenses sold (r3: β_3yr_CWD_ = −0.00 ± 0.03; *p* = 0.95).

### Robustness checks

**FIGURE 5:**
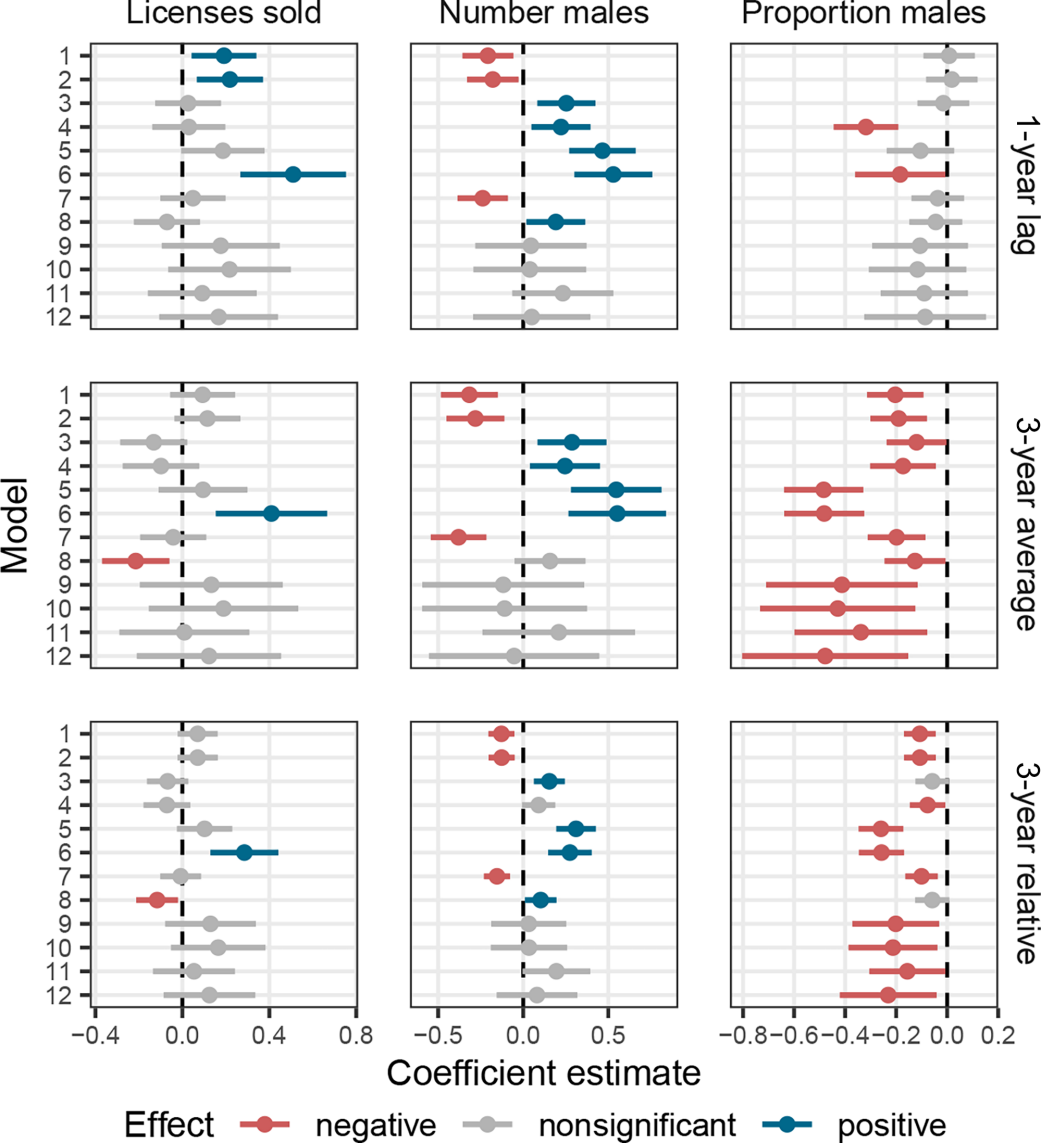
Coefficient estimates (mean and 95% CI) from 12 different model structures (Appendix S1: Table S3), estimating the effects of harvest on chronic wasting disease (CWD) prevalence. Harvest metrics were *z*‐transformed. The number of licenses sold had an inconsistent or non‐significant relationship with prevalence. The absolute number of males harvested was also inconsistent, with some models estimating a significantly negative effect of harvest and others estimating a significantly positive effect. When the proportion of males harvested was used, coefficient estimates were robust across all models (all models predicted a negative effect of proportion of males harvested), although the significance and magnitude varied. Harvest pressure at a 1‐year lag (top row) was generally not a significant predictor of subsequent CWD prevalence, relative to the 3‐year mean (middle row) or the 3‐year relative hunting pressure (i.e., the difference in the hunting pressure relative to a herd's mean; bottom row). For models with interactions only, the main effect (the effect of harvest at the mean year) is plotted.

The number of licenses sold and the number of males harvested had inconsistent relationships with prevalence, with some models estimating a positive effect and others a negative effect (Figure 5). However, the proportion of males harvested had a negative effect on CWD prevalence that was robust across model formulations, especially when 3‐year lags were used. While the proportion of males harvested was consistently associated with a reduction in prevalence, we caution against making precise predictions of prevalence in response to a specific change in harvest, due to how the effect size varied across model structures (Figure 5) and when uncertainty in covariates was included (Appendix S2).

## DISCUSSION

Identifying a causal effect of harvest on disease transmission in wild populations is difficult because harvest is rarely applied using an experimental approach and because its relationship with disease may involve feedbacks and confounding variables. Thus, while disease transmission may be affected by harvest, high disease prevalence may also affect population sizes and harvest rates (Figure 2). We applied a combination of statistical modeling approaches to a long‐term dataset from Wyoming mule deer herds to more clearly estimate the effects of harvest on CWD prevalence, both spatially (i.e., across herds) and temporally (i.e., within herds). Our results demonstrate that herds with consistently high hunting pressure on males across more than 20 years had lower CWD prevalence (although in no cases was CWD eradicated). Moreover, increasing the relative harvest pressure within a herd over a shorter time period is associated with reduced CWD prevalence, albeit to a smaller degree. Altogether, our results suggest an important role for maintaining consistently high harvest pressure on adult males after CWD has entered a population, corroborating the effectiveness of management interventions that are already being implemented or actively encouraged across North America (Thompson et al., 2023; Western Association of Fish and Wildlife Agencies, 2017).

We observed a consistently negative relationship between CWD prevalence and harvest pressure, especially the proportion of males harvested (Figure 5). In the absence of experimental data, it is difficult to conclusively prove the causal role of harvest; however, several lines of evidence support our interpretation of harvest as a causal driver. While a correlation between mean harvest pressure and CWD prevalence across herds could potentially be driven by confounding variables (e.g., habitat quality, population structure, geographic location) that affect both harvest rates and CWD transmission, we found a negative effect of harvest when using the relative harvest pressure within a herd, essentially controlling for these differences across herds (Genser et al., 2015). Moreover, reductions in harvest rates within a herd were associated with higher subsequent prevalence, yet the reverse was not true (higher prevalence did not necessarily lead to lower harvest rates), which suggests that a change in harvest is the driving variable. The negative effect of harvest (proportion of males harvested) was also robust across different forms of model structure (Figure 5), including those that controlled for herd‐level effects, temporal trends, and demographic variables (Appendix S1: Table S3)—indicating that links between harvest and prevalence are unlikely to be due to confounders or reverse causality (Addicott et al., 2022; Oster, 2019). Although the effect of male harvest pressure was consistently negative, its magnitude was less certain. The effect size varied across model formulations (Figure 5) and included relatively wide confidence intervals (Figure 4). Under these current estimates, we are unable to predict the effect of a given change in harvest with a high degree of precision.

Our analysis demonstrates the importance of carefully selecting appropriate metrics that capture relevant aspects of harvest pressure. For example, the total number of males harvested and the number of licenses sold, both used in previous analyses (Conner et al., 2021; Miller et al., 2020), produced inconsistent estimates, even switching from a positive to negative effects depending on the model structure used (Figure 5). It is possible that these variables are confounded with others or that they simply do not have an influence on prevalence. For instance, the number of licenses sold, while a metric that is manipulable by managers, may not correspond with actual harvest pressure experienced by populations (Brandell et al., 2022). Indeed, the number of licenses sold was only weakly correlated with the proportion of males harvested (*r* = 0.31). It is also perhaps unsurprising that harvest pressure scaled by population size (e.g., *proportion* of males harvested) is a more meaningful estimate of harvest pressure than the absolute number of males harvested, which may be confounded by variables like herd size that could influence (or be influenced by) CWD. Standardizing harvest estimates by population size appears to be key but currently, not all agencies estimate population abundance or relevant harvest metrics (Brandell et al., 2022; Conner et al., 2021). Equally important is testing the impact of harvest across relevant temporal windows. Because individuals do not recover from CWD, prevalence in adults is determined by risk across those individuals' entire lifetimes. Consequently, harvest in the year prior is unlikely to be a strong predictor of prevalence in the following year. We chose an overall time‐lag roughly equal to the average age or exposure time of the population. However, if age information is collected alongside CWD status (which was not the case in the current dataset), then individuals’ varying windows of exposure could be explicitly accounted for in the modeling approach (Heisey et al., 2010).

As more agencies implement CWD management and surveillance plans, there will likely be increased interest in rigorously evaluating the impact of interventions using longitudinal data. In addition to the selection of appropriate time lags and harvest metrics (above), we believe the following analytical guidelines will help better isolate the effects of harvest on disease prevalence. An autoregressive model or the inclusion of calendar time (i.e., year) in the model helps account for a potential feedback between disease prevalence and harvest, as well as potential confounding temporal trends (Figure 2). Investigating the spatial and temporal variation in harvest separately using average and relative harvest levels helps assess the degree of control that a short‐term management intervention could have and is more robust to spatial confounders. While feedbacks between CWD prevalence and relative harvest pressure were not observed in our study, it is clear that feedbacks between management intensity and prevalence may exist in other jurisdictions (Thompson et al., 2023) or for other wildlife disease systems. Testing whether disease is correlated with lower harvest or population size at some future time provides more confidence in the directionality of harvest impacting disease, rather than the reverse. While retrospective analyses using observational data will likely become increasingly common, we also emphasize that implementation of harvest using an experimental design (e.g., Donnelly et al., 2006) would provide even more valuable information on the causal role of harvest and the magnitude of its effects. Experimental approaches are undoubtedly politically and logistically challenging, especially over longer timeframes, but are likely to be worthwhile as the relevance and demand for science on wildlife disease management grows.

There are several potential mechanisms through which harvest could alter disease prevalence (Figure 1). Our analysis was not intended to discriminate between these mechanisms, although our findings were suggestive that the effects of harvest do not solely operate through reductions in density or shifts in male–female ratio. Other analytical approaches, such as structural equation modeling or mechanistic disease modeling, could be leveraged to better understand the pathways through which harvest affects prevalence. An understanding of mechanism is crucial because some mechanisms slow transmission directly while others rely upon significantly shifting population structure, which have different implications for optimal management strategy and long‐term predictions of population and disease trends. For example, even in populations where low prevalence is achieved by removing older (infected) males, there may still be high transmission risk due to environmental contamination (Almberg et al., 2011). Relatedly, given limited data from other age–sex classes, we could not test whether male‐based harvest strategies that reduce prevalence in males also alter disease risk to females or juveniles, which is important for understanding transmission and its influence on population dynamics. We also did not estimate how harvest pressure and prevalence varied across ages, although doing so could provide insights on how to optimize harvest strategies for CWD control. An understanding of mechanism would also help inform whether other sources of mortality (e.g., high mortality due to severe winters or other disease outbreaks) are functionally equivalent to harvest—a key question for agencies deciding whether to limit harvest after population perturbations. Insight into mechanism will likely require even larger datasets, perhaps achieved by combining information from multiple jurisdictions (e.g., Conner et al., 2021) and devoting significant effort to the collection of age‐ and sex‐structured surveillance data. More broadly, gaining a mechanistic understanding of the role of harvest in wildlife disease transmission will require comparative analyses across disease systems that vary in harvest strategy, disease transmission, and population biology and will benefit from the integration of theory, experimentation, and observational data (Joseph et al., 2013).

While our results are indicative of a role for harvest in mitigating CWD prevalence, there are numerous practical considerations in implementing harvest‐based strategies to control CWD. Herds with higher harvest pressure over long time periods had lower prevalence, whereas changing harvest over short time periods (i.e., 3 years) had relatively small, yet still measurable effects. We caution that a single, short‐term intervention is unlikely to reshape the trajectory of CWD in a population, especially if applied later in the epidemic when environmental transmission or prevalence in females may be high (Almberg et al., 2011; Uehlinger et al., 2016). Instead, our findings are in line with previous work suggesting that removal‐based strategies are most effective when implemented on relatively long time scales (Conner et al., 2021; Geremia et al., 2015; Prentice et al., 2019; Wasserberg et al., 2009). Maintaining consistently high harvest pressure on adult males can be feasible from a population sustainability perspective (Jennelle et al., 2014; Rogers et al., 2022) but must be balanced with other objectives, such as maintaining recreational opportunities and stakeholder support (Cook et al., 2023; Heberlein, 2004). Thus, even if high harvest rates are effective at limiting CWD, achieving those high harvest rates across multiple decades with hunter participation is not straightforward and could entail tradeoffs that are undesirable to the public or to management agencies. Importantly, CWD was still present even in herds with the highest levels of harvest pressure (40% of males removed each year, on average), and high harvest pressures in our study populations primarily appeared to drive a slower rate of increase, rather than a decrease in prevalence over time (Appendix S1: Figure S5). This suggests that few herds will have the ecological or social conditions to support the levels of harvest needed to eradicate the disease. Overall, our results suggest that maintaining hunting pressure after CWD is detected can have measurable impacts on disease prevalence (and has the additional benefit of providing important surveillance data), but it requires realistic expectations.

As novel wildlife diseases increasingly emerge, natural resource agencies require practical, evidence‐based strategies to manage disease. Harvest‐based strategies can be controversial, difficult to implement, and do not always produce the intended outcome of limiting disease spread, but despite these challenges, they are still often recommended for disease control. In addition to theoretical and simulation‐based models, observational evidence is needed to understand where and when harvest is an effective tool and the magnitude of its effects. Herein, we have leveraged a long‐term surveillance dataset and causally informed statistical techniques to provide robust evidence on the effects of recreational harvest for limiting a particularly challenging wildlife disease. Although harvest is unlikely to eradicate disease on its own, when applied with other disease control measures (e.g., reducing aggregation or long‐distance spread), it may be an effective method for dampening the epidemic curve.

## AUTHOR CONTRIBUTIONS

All authors contributed to project conceptualization. Samantha E. Allen, Justin Binfet, William H. Edwards, Jessica E. Jennings‐Gaines, and L. Embere Hall supported or oversaw the data collection and collation. Wynne E. Moss and Paul C. Cross analyzed the data. Wynne E. Moss led the manuscript writing. All authors provided feedback on the manuscript.

## CONFLICT OF INTEREST STATEMENT

The authors declare no conflicts of interest.

## Supporting information


Appendix S1.

Appendix S2.

## Data Availability

Data and code (Moss, 2024) are available from Zenodo: https://doi.org/10.5281/zenodo.14047105.
