# Supplementary material for: The effectiveness of harvest for limiting wildlife disease: Insights from 20 years of chronic wasting disease in Wyoming
Source: Ecol Appl. 2025 Jan 21;35(1):e3089. doi: 10.1002/eap.3089 (PMC11748107; doi:10.1002/eap.3089)
Supplement: Supplementary file 1 — Appendix S1. [file EAP-35-e3089-s001.pdf]

Appendix S1

Title: The effectiveness of harvest for limiting wildlife disease: Insights from 20 years of chronic wasting disease in Wyoming

Authors: Wynne E. Moss, Justin Binfet, L. Embere Hall, Samantha E. Allen, William H. Edwards, Jessica E. Jennings-Gaines, Paul C. Cross

Journal: Ecological Applications

Supplemental Methods

Covariates

Here, we describe methods used by Wyoming Game and Fish Department (WGFD) to produce estimates of population size, demographic ratios, and harvest rates. These methods were employed during the course of our study (2000 – 2021) but may not reflect current practices used by WGFD.

Estimates of demographic ratios (buck:doe and fawn:doe ratios) were derived annually for each herd, following the hunting season. To determine ratios, biologists observed groups of animals on winter range, usually November-December, and recorded the number of individuals in each age and sex cohort. Observations were made either from the ground or from the air. Sampling effort (number of groups observed) was guided by herd-specific precision targets in an attempt to limit the margin of error (80% confidence interval) on the estimate to below 5 animals per 100 does. On average, 1994 individuals were counted in each herd unit in a year (range: 283 - 7941). Confidence intervals for demographic ratios were estimated using the methods described in Czaplewski et al. (1983). The number of bucks and fawns in each herd was calculated relative to 100 does.

Population sizes were estimated using a Bayesian integrated population model (IPM; see Nowak et al. 2018 for additional detail). Briefly, the IPM used multiple streams of data to produce an estimate of total population size for each herd in each year. Data included estimates of harvest (all years), population ratios (all years), vital rates (when available), and estimated abundances from detection-corrected surveys (when available).

A density estimate (deer/km^2) was computed by dividing the annual population estimate by the area potentially used by the herd during the winter months (i.e., winter range). Winter range was a time-invariant covariate derived from WGFD’s seasonal range maps (Wyoming Game and Fish Department 2021). For each herd, seasonal range boundaries are mapped based upon multiple decades of observations from local biologists and game wardens, research efforts, and data from tagging and radio collar studies (Wyoming Chapter of the Wildlife Society 1990, Wyoming Game and Fish Department 2002). We considered ‘winter range’ to be any area potentially used by individuals during the winter months, which excluded areas used only in spring, summer, or fall and those designated as non-habitat. Dividing by winter range provided an estimate of density during the season in which individuals are most concentrated.

We used information on annual harvest rates and license issuance from WGFD’s harvest reports (https://wgfd.wyo.gov/Hunting/Harvest-Reports). Wyoming Game and Fish Department offers a variety of licenses, some of which cover a general area encompassing several herds (general licenses; Table S2). The Department tallies the number of licenses sold each year, and a harvest survey is used to estimate the number of active licenses and deer harvested within each herd unit. As estimates of harvest pressure (Table S2), we used number of adult males harvested (*totalmale*), licenses sold (*licensesold*). For the purpose of this study, ‘adult male’ is a yearling or older. We also calculated the proportion of males harvested (*propmale*) using both the harvest information and demographic estimates and the following formulae:

$$(1)\ propmale = \frac{number\ males\ harvested}{total\ males\ postharvest + number\ males\ harvested}$$

$$(2)\ total\ males\ postharvest = total\ females\ postharvest\ \times male{:}female\ ratio$$

$$(3)\ total\ females\ postharvest = \frac{100 \times population\ size}{male{:}female\ ratio + juvenile{:}female\ ratio + 100}$$

Because several covariates used in our model involve measurement error, we used simulations to investigate the effects of measurement error on parameter estimates (Appendix S2).

Data analysis

Statistical analyses focused on estimating the effects of harvest on CWD prevalence, with the hypothesis that higher rates of harvest in previous years would negatively affect CWD prevalence in the year *t*. We fit a series of models (Table S2) to control for various issues that impact causal inference and estimation of the focal relationship. Model parameterization was based upon knowledge of potential relationships among variables with the aim to reduce the influence of confounding variables (Main text: Figure 2).

First, to account for the confounding effect of previous years' CWD prevalence (Figure 2), which could be driven by feedbacks between CWD prevalence and harvest rates, we utilized autoregressive models (Table S2; models a1–a5). This model controlled for previous years' prevalence, blocking a backdoor path between previous prevalence and current prevalence. We also directly tested the potential feedback between CWD prevalence and subsequent harvest rates (i.e., the potential for reverse causality; Figure 2) using linear models where lagged CWD prevalence was the predictor variable and harvest rates were the response variable (Table S2; models r1 – r3) .

During the early stages of CWD epidemics (which can last decades) CWD prevalence often increases through time. Concurrently, harvest rates have declined through time in Wyoming and other jurisdictions (Figure S1), for a variety of reasons including declining hunter participation and population declines. Therefore, harvest rates and annual estimates of CWD prevalence may have opposite temporal trends, and may be negatively correlated with one another, without a true causal relationship (Figure 2). We fit multiple models that included year as a covariate to better control for the confounding effect of time (Table S3) and also used proportion of males harvested as a covariate, since it did not show a temporal trend (Figure S1).

Certain metrics of harvest may be affected by confounders like population size, year, or previous CWD prevalence. For instance, the total number of males harvested could be influenced by herd size, which might also impact CWD dynamics (Figure 2). Similarly, the mean harvest pressure within a herd could be confounded with other herd-level, spatial confounders, such as habitat quality, land use or spatial orientation (which could also affect CWD transmission or epidemic history). We used standardized (e.g., proportion of bucks harvested, active licenses per deer) and relativized (e.g., 3-year relative harvest pressure, compared to the herd's average) harvest metrics to remove some of these potential confounders. For example, the relative harvest within a herd in a given three-year period (i.e., the deviation in harvest pressure) should not be related to spatial confounders. Thus, including this variable standardizes for differences across herds and emphasizes the effects of harvest within herds, which improves the robustness of causal inference (Genser et al. 2015, Byrnes and Dee 2024). We also addressed the potential for density and buck:doe ratio to be confounders or mediators by including these as predictor variables in the autoregressive model (model a5).

We acknowledge that there may be other unknown and unaccounted for confounding variables or feedback mechanisms. We fit a series of additional models (Table S3) varying in their model form to test whether the effects of harvest were consistent across model form. Stability in coefficient estimates, especially the sign of the coefficient, indicates that the effect of harvest is not sensitive to the inclusion of certain controls or omitted variables. Robustness of estimate across model structure is therefore a key component of making causal claims about the effect of a parameter (Oster 2019, Addicott et al. 2022).

References

Addicott, E. T., E. P. Fenichel, M. A. Bradford, M. L. Pinsky, and S. A. Wood. 2022. Toward an improved understanding of causation in the ecological sciences. Frontiers in Ecology and the Environment 20:474–480.

Byrnes, J. E. K., and L. E. Dee. 2024. Causal inference with observational data and unobserved confounding variables. Preprint, bioRxiv. https://doi.org/10.1101/2024.02.26.582072.

Czaplewski, R. L., D. M. Crowe, and L. McDonald. 1983. Sample Sizes and Confidence Intervals for Wildlife Population Ratios. Wildlife Society Bulletin 11:121–128.

Genser, B., C. A. Teles, M. L. Barreto, and J. E. Fischer. 2015. Within- and between-group regression for improving the robustness of causal claims in cross-sectional analysis. Environmental Health 14:60.

Hartig, F. 2022. DHARMa: Residual Diagnostics for Hierarchical (Multi-Level/Mixed) Regression Models. R package version 0.4.6. https://CRAN.R-project/org/package=DHARMa.

Nowak, J. J., P. M. Lukacs, M. A. Hurley, A. J. Lindbloom, K. A. Robling, J. A. Gude, and H. Robinson. 2018. Customized software to streamline routine analyses for wildlife management. Wildlife Society Bulletin 42:144–149.

Oster, E. 2019. Unobservable Selection and Coefficient Stability: Theory and Evidence. Journal of Business & Economic Statistics 37:187–204.

Wyoming Chapter of the Wildlife Society. 1990. Appendix 6A: Standardized Definitions for Seasonal Wildlife Ranges *in* Wildlife Handbook of Biological Techniques. Wyoming Game and Fish Department, Cheyenne, Wyoming, USA. https://wgfd.wyo.gov/sites/default/files/content/PDF/Wildlife/Handbook-BioTechniques/A06a-DefSeasRange.pdf

Wyoming Game and Fish Department. 2002. Appendix 6B: Revising herd unit seasonal range maps and migration corridors *in* Wildlife Handbook of Biological Techniques. Wyoming Game and Fish Department, Cheyenne, Wyoming, USA. https://wgfd.wyo.gov/sites/default/files/content/PDF/Wildlife/Handbook-BioTechniques/A06b-ReviseSeasRange.pdf

Wyoming Game and Fish Department. 2021. Mule Deer Seasonal Range Boundaries. Shapefile. https://services6.arcgis.com/cWzdqIyxbijuhPLw/arcgis/rest/services/MuleDeerSeasonalRange/FeatureServer

Supplemental Tables

Table S1. For each of the focal mule deer (*Odocoileus hemionus*) herds analyzed in this study, we report the area occupied, density, harvest information, and chronic wasting disease prevalence (proportion of adult males infected), averaged across the years of the study (2000 - 2021).

				Harvest				Chronic wasting disease		
**Herd**	**Winter range (km^2)**	**Total range (km^2)**	**Density ($deer/km^2$)**	**Proportion males harvested**	**Active license per deer**	**Season structure***	**Strategy†**	**Number tested**	**Number positive**	**Prevalence**
South Converse	1,927	3,165	4.52	0.20	0.12	G	PL	658	250	0.38
Goshen Rim	13,676	13,715	1.15	0.22	0.13	G	R	763	231	0.30
Platte Valley	4,190	6,335	4.46	0.23	0.14	LQ	R	1683	56	0.03
Laramie Mountains	8,602	12,430	2.37	0.23	0.11	G, LQ	R	2653	666	0.25
Sheep Mountain	4,649	6,420	2.15	0.23	0.21	G	R	1123	77	0.07
Southwest Bighorn	6,892	7,630	2.65	0.26	0.15	G, LQ	R	1918	83	0.04
Upper Powder River	2,633	3,578	4.77	0.27	0.15	G	S	2024	99	0.05
Bates Hole/Hat Six	1,888	2,660	3.44	0.28	0.16	G	S	568	127	0.22
Baggs	6,710	8,678	2.99	0.33	0.16	G, LQ	S	2651	73	0.03
Black Hills	7,232	8,139	3.35	0.40	0.26	G	R	755	19	0.03

*Season structures include: G = general license (unlimited resident licenses); LQ = limited quota (limited licenses)

†Management strategies include: R = recreational (20 - 29 bucks:100 does); S = special (30-45 bucks:100 does); PL = private land (limited WGFD influence due to low access)

Table S2. Summary of statistical models used. We ran three general types of statistical models to address specific issues regarding confounding variables or potential feedbacks (Figure S1), with the general goal of understanding the effects of harvest (measured using multiple metrics) on chronic wasting disease (CWD) prevalence.

Analysis	Purpose	Model form(s)	Main findings
Autoregressive model	Account for non-linearity, temporal autocorrelation, and feedbacks between harvest and CWD	a1: CWD_t ~ mean_propmale + 3yr_rel_propmale + AR1 + (1|herd)<br>a2: CWD_t ~ mean_totalmale + 3yr_rel_totalmale + AR1 + (1|herd)<br>a3: CWD_t ~ mean_activelicenseperdeer + 3yr_rel_activelicenseperdeer + AR1 + (1|herd)<br>a4: CWD_t ~ mean_licensesold + 3yr_rel_licensesold + AR1 + (1|herd)	• Negative effect of *propmale* (mean and relative pressure) on CWD<br>• Other harvest metrics non-significant or marginally significant.<br>• *Density* and *buck:doe* ratio not significant predictors of CWD prevalence
	Test for mediating effects of density and demographic structure	a5: CWD_t ~ mean_propmale + 3yr_rel_propmale + $density_{t-1}$ + $buck{:}doe_{t-1}$ + AR1 + (1|herd)	
Reverse causality	Test whether CWD affected subsequent harvest pressure	r1: $rel\_propmale_t$ ~ 3yr_CWD + year + (1|herd)<br>r2: $rel\_activelicenseperdeer_t$ ~ 3yr_CWD + year + (1|herd)<br>r3: $rel\_licensesold_t$ ~ 3yr_CWD + year + (1|herd)	• No effect of CWD on relative harvest pressure
Robustness check	Evaluate whether effects of harvest on CWD are consistent across model structure	see Table S3	• Negative effect of *propmale* is consistent across models

Table S3. Models fit to evaluate robustness. We fit 12 different types of models varying in covariates used and model structure. For each different form, we substituted one of nine potential metrics of harvest pressure, for a total of 108 models. Random intercepts are represented using a (1|) symbol (i.e., (1|herd) denotes a random intercept term for each herd). Random slopes (i.e., a different temporal trend for each herd) are denoted as (year|herd). Auto-regressive terms are represented with AR1.

**Model forms**	
Model	*Formula*
1	~[harvest pressure] + (1|herd)
2	~[harvest pressure] + herd
3	~[harvest pressure] + year + (1|herd)
4	~[harvest pressure] × year + (1|herd)
5	~[harvest pressure] + year + (year|herd)
6	~[harvest pressure] × year + (year|herd)
7	~[harvest pressure] + CWD_{t-1} +(1|herd)
8	~[harvest pressure] + CWD_{t-1} + year + (1|herd)
9	~[harvest pressure] + (1|herd) + AR1
10	~[harvest pressure] + CWD_{t-1} + (1|herd) + AR1
11	~[harvest pressure] + year + (1|herd) + AR1
12	~[harvest pressure] + $buck{:}doe_{t-1}$ + $density_{t-1}$ +(1|herd) + AR1

**Harvest pressure metrics**	
totalmale	Number of males harvested (prior year)
	Number of males harvested (prior three years)
	Number of males harvested (prior three years) relative to herd average*
propmale	Proportion of males harvested (prior year)
	Proportion of males harvested (prior three years)
	Proportion of males harvested (prior three years) relative to herd average*
licensesold	Number of licenses sold (prior year)
	Number of licenses sold (prior three years)
	Number of licenses sold (prior three years) relative to herd average*

*Relative harvest pressure was determined by taking the prior three year average and subtracting the herd's mean

Table S4. Parameter estimates reflecting the effect of harvest on CWD prevalence, for autoregressive models. We present estimates from models in which overly influential points were removed.

Model	Parameter	Estimate	SE	Z	P value
a1	Intercept	1.44	1.82	0.80	0.43
	3yr_rel_propmale	-4.16	2.13	-1.96	0.05
	mean_propmale	-14.9	6.77	-2.21	0.03
a2	Intercept	-0.77	0.88	-0.88	0.38
	3yr_rel_totalmale	0.16	0.53	0.31	0.76
	mean_totalmale	-2.01	0.89	-2.26	0.02
a3	Intercept	-0.16	1.70	-0.09	0.93
	3yr_rel_activelicenseperdeer	-5.74	3.42	-1.68	0.09
	mean_activelicenseperdeer	-14.9	10.4	-1.44	0.15
a4	Intercept	-1.89	0.59	-3.20	0.00
	3yr_rel_licensesold	0.81	0.46	1.78	0.07
	mean_licensesold	-1.66	1.06	-1.57	0.12
a5	Intercept	1.54	1.82	0.85	0.40
	3yr_rel_propmale	-4.95	2.48	-2.00	0.05
	mean_propmale	-15.2	6.51	-2.33	0.02
	density(t-1)	-0.11	0.11	-1.00	0.32
	buck:doe(t-1)	0.01	0.01	0.82	0.41

Supplemental Figures

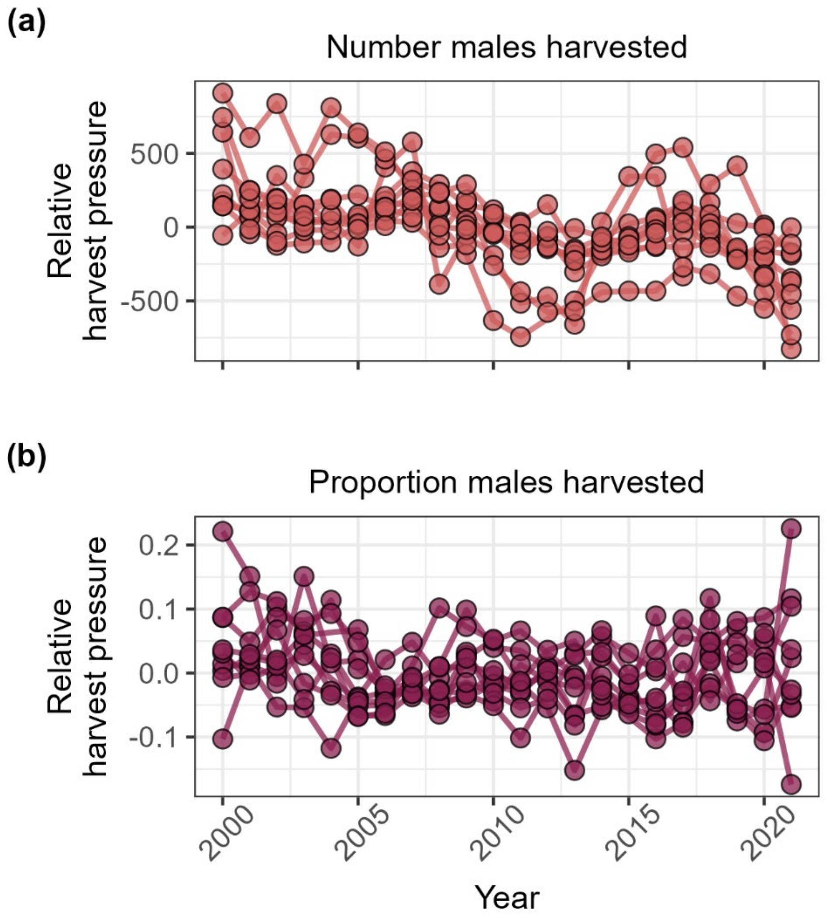

Figure S1 (a) Trends in the number of adult males harvested per year and (b) proportion of adult males harvested per year, with each line representing one of ten mule deer herds. To standardize for differences across herds, the relative harvest pressure is shown (i.e., each year's harvest pressure was compared to the 20-year mean for that herd).

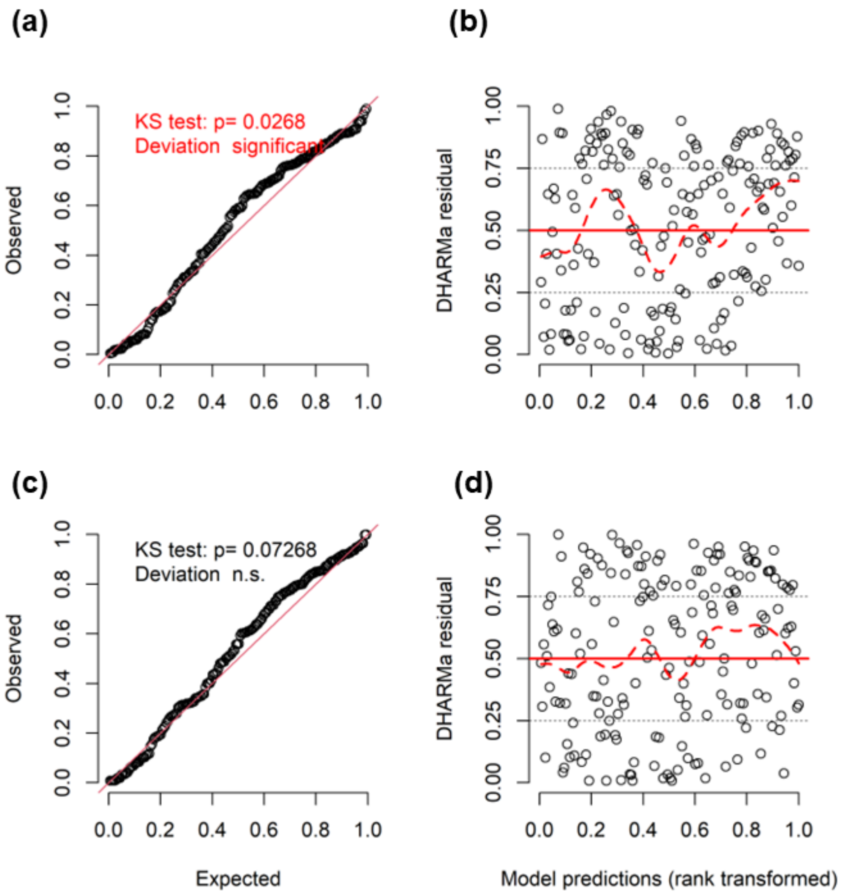

Figure S2. Model diagnostics for models a1 and a5, which were autoregressive models that either excluded (model a1; panels a-b) or included (model a5; panels c-d) demographic covariates (see Table S2). Left panels show QQ plots and the results from a test for uniformity. Right panels show the relationship between model predictions and residuals. Model diagnostics were performed using the package DHARMa (Hartig 2022) which simulates residuals for non-normal response variables. Tests performed in DHARMa (testResiduals function) indicated no significant issues with outliers or dispersion.

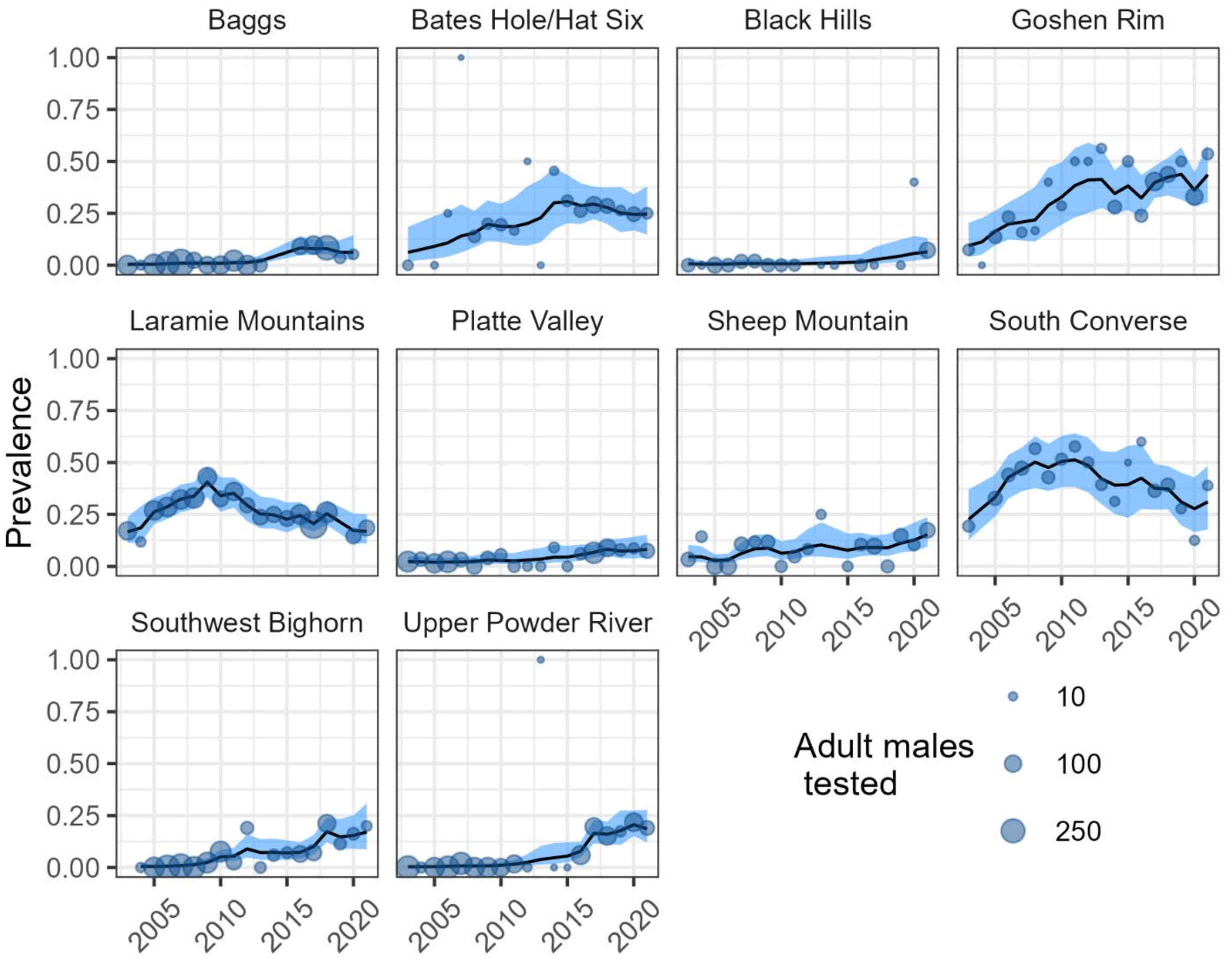

Figure S3. Model fit for the autoregressive model (a5; see Table S2. The predicted prevalence and 95% confidence interval from the model is depicted by the ribbon, with observations overlaid.

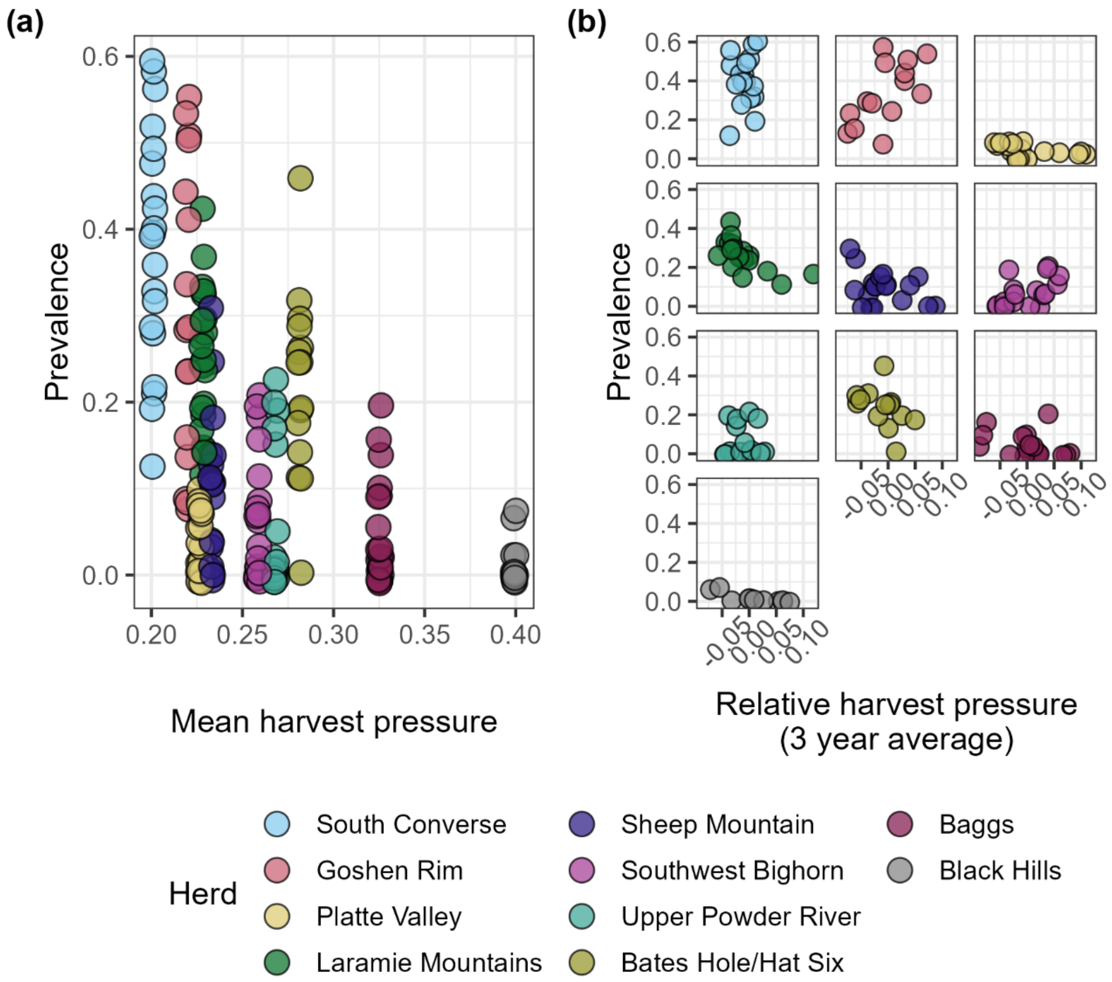

Figure S4. (a) The relationship between chronic wasting disease prevalence and mean harvest pressure in 10 mule deer herds in Wyoming. Herds with higher harvest pressure (proportion of males harvested) across the entire study period (2000 – 2021) had lower prevalence. (b) Within herds, years with higher harvest pressure relative to that herd's mean were less clearly associated with lower prevalence but still significantly related.

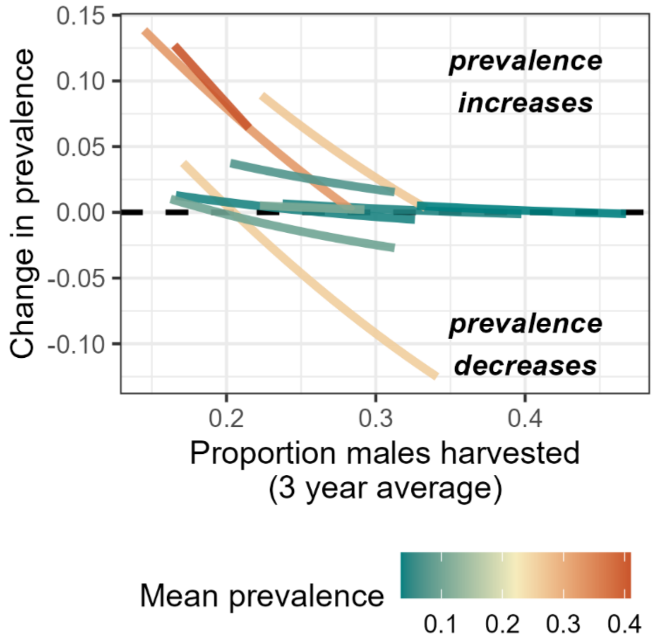

Figure S5. Predicted effect of a short-term (three-year) change in harvest in 10 different mule deer herds. For each herd, we show the predicted change in CWD prevalence from year 9 to year 10, across the range of harvest pressures experienced by that herd. At low levels of harvest, prevalence is expected to increase; however, this rate of increase is attenuated as harvest pressure increases, with sufficiently high harvest rates leading to declines in prevalence from one year to the next. The level of male harvest pressure needed to reduce prevalence varies by herd, in part because the model uses relative harvest pressure (compared to each herd's average) rather than absolute harvest pressure.
