# Supplementary material for: The effectiveness of harvest for limiting wildlife disease: Insights from 20 years of chronic wasting disease in Wyoming
Source: Ecol Appl. 2025 Jan 21;35(1):e3089. doi: 10.1002/eap.3089 (PMC11748107; doi:10.1002/eap.3089)
Supplement: Supplementary file 2 — Appendix S2. [file EAP-35-e3089-s002.pdf]

Appendix S2: Exploration of Covariate Uncertainty

Title: The effectiveness of harvest for limiting wildlife disease: Insights from 20 years of chronic wasting disease in Wyoming

Authors: Wynne E. Moss, Justin Binfet, L. Embere Hall, Samantha E. Allen, William H. Edwards, Jessica E. Jennings-Gaines, Paul C. Cross

Journal: Ecological Applications

Appendix S2: Exploration of Covariate Uncertainty

In this analysis, we investigate how uncertainty in model covariates affects parameter estimation. We do so by simulating new datasets, for which we add random (measurement) error in the covariates. The amount of measurement error for each covariate is estimated using available data from the Wyoming Game & Fish Department. After simulating a new dataset, we then fit a linear model and extract the parameter estimates. We repeat for 100 simulated datasets to understand the stability of the parameter estimate.

We investigate measurement error in four estimates, which are used to calculate model covariates. Wyoming Game and Fish Department annually conducts surveys to estimate the following variables (Appendix S1: Supplemental Methods):

(1) *Population size*, estimated using population surveys conducted post-harvest and an integrated population model (IPM);

(2) *Male:female ratio*, estimated using annual counts conducted post-harvest;

(3) *Juvenile:female ratio*, estimated using annual counts conducted post-harvest;

(4) *Total males harvested*, estimated using a harvest survey.

These variables are then used to calculate the total (pre-harvest) number of males in the population and the proportion of males that were harvested in a given year (*propmale*; see Appendix S1: Supplemental Methods).

Here, we consider how observation error in the four variables above contributes to overall observation error in the covariate *propmale* and the estimation of the parameter of interest ($\beta_{rel\_propmale\_3YA}$: the effect of male harvest pressure on CWD prevalence). Estimates of uncertainty in the four variables were available for all 10 herds only in recent years (2021, 2022, or 2023). These were provided as 90 or 95% confidence intervals.

Estimating Observation Error

Observation Error for Population Size:

Population size estimates with uncertainty were obtained from an IPM and full outputs were available for 2023 only. The IPM represents uncertainty using 95% posterior credibility intervals. We make the assumption that these are similar to a 95% confidence interval on the estimate of population size. Estimates with uncertainty are shown in Table S2.1. We solved for $\sigma_{\bar{X}}$ or the standard deviation of the sample mean using the formula for a 95% confidence interval for a normally distributed variable: $CI = \bar{X} \pm 1.96 * \sigma_{\bar{X}}$, where $\sigma_{\bar{X}}$ is an estimate of observation error. This generated an estimate of uncertainty in estimated population size for each herd.

Table S1. Estimated population sizes and 95% credibility intervals for mule deer herds, produced by integrated population models.

Herd	Year	Post-season population size	95% credibility interval
Baggs	2023	11833	9997 - 13827
Bates Hole/Hat Six	2023	3707	3301 - 4106
Black Hills	2022	13194	12183 - 14440
Goshen Rim	2023	6112	5326 - 7148
Laramie Mountains	2023	6968	6132 - 7892
Platte Valley	2023	12455	10803 - 14249
Sheep Mountain	2023	3416	3049 - 3854
South Converse	2023	3883	3037 - 4895
Southwest Bighorn	2023	8443	7348 - 9620
Upper Powder River	2023	5150	4680 -5783

Observation Error for Harvest Rate:

Measurement error for male harvest was obtained from the 2022 Harvest Report. Uncertainty was reported as 90% confidence intervals (Table S2). Again, using the 90% confidence interval, we solved for $\sigma_{\bar{x}}$ or the standard deviation of the sample mean using the formula: $CI = \bar{x} \pm 1.645 * \sigma_{\bar{x}}$, where $\sigma_{\bar{x}}$ is an estimate of observation error.

Table S2. Estimated male harvest (number adult males harvested) and 90% confidence intervals for mule deer herds.

Herd	Year	Male harvest	90% confidence interval
Baggs	2022	321	268 - 374
Bates Hole/Hat Six	2022	400	347 - 453
Black Hills	2022	987	890 - 1084
Goshen Rim	2022	487	426 - 548
Laramie Mountains	2022	680	610 - 750
Platte Valley	2022	312	268 - 356
Sheep Mountain	2022	342	287 - 397
South Converse	2022	221	177 - 265
Southwest Bighorn	2022	401	352 - 450
Upper Powder River	2022	518	457 - 579

Observation Error for Demographic Ratios

Uncertainty on demographic ratios (male:female ratio and juvenile:female ratio) were obtained from WGFD for almost all years. Uncertainty was reported as a margin of error (MOE) for a 90% confidence interval ($MOE = 1.645 * \sigma_{\bar{X}}$). We used the most recent year available for each herd (2022 for 9 herds, 2021 for Laramie Mountains; Table S3). We solved for $\sigma_{\bar{X}}$ or the standard deviation using the same formula as above.

Table S3. Male:female ratio, juvenile:female ratio and associated margin of error (MOE) for mule deer herds.

Herd	Year	M:F ratio	MOE	J:F ratio	MOE
Southwest Bighorn	2022	30.4	4.4	60.8	6.9
Upper Powder River	2022	22.1	2.9	56.8	5.2
Baggs	2022	27.4	1.5	65.2	2.5
Goshen Rim	2022	30.0	5.3	44.8	6.7
Laramie Mountains	2021	33.6	6.0	41.1	6.8
Sheep Mountain	2022	26.6	4.6	58.3	7.5
Platte Valley	2022	33.8	5.7	76.2	9.8
Black Hills	2022	16.7	2.4	55.0	4.9
South Converse	2022	41.1	9.1	74.3	13.5
Bates Hole/Hat Six	2022	30.4	6.1	65.4	9.9

Overall Observation Uncertainty

We combined the above into a single table representing observation uncertainty ($\sigma_{\bar{X}}$) in all 4 of the variables, for each herd (Table S4).

Table S4. Estimated observation error (standard deviation) in four variables, for each of 10 mule deer herds.

Herd	Population size	Male harvest	Male:female ratio	Juvenile:female ratio
Baggs	977.0	32.2	0.9	1.5
Bates Hole/Hat Six	205.4	32.2	3.7	6.0
Black Hills	575.8	59.0	1.4	3.0
Goshen Rim	464.8	37.1	3.2	4.1
Laramie Mountains	449.0	42.6	3.7	4.1
Platte Valley	879.1	26.7	3.5	6.0
Sheep Mountain	205.4	33.4	2.8	4.6
South Converse	474.0	26.7	5.6	8.2
Southwest Bighorn	579.6	29.8	2.7	4.2
Upper Powder River	281.4	37.1	1.8	3.1

Simulating covariates

We simulated estimates of population ratios, population size, and male harvest, using the observation uncertainty estimated above. We assumed here that uncertainty in covariate estimates varies across herds (Table S4), but is constant across time (i.e., the observation error does not change systematically over time). To represent measurement error in the covariates, for each simulation run, we added a normally distributed error with mean of 0 and a standard deviation as estimated above. For example, for the herd Baggs, the four different standard deviations (see table above) were used to draw simulated errors in male:female ratio, juvenile:female ratio, population size, and male harvest for each year. Adding these errors to the estimates in the dataset produces a simulated estimate ('SIM') of all four variables in each year (e.g., Table S5).

These simulated variables were then used to calculate proportion of males harvested (*propmale*) using the formulae outlined in Appendix S1: Supplemental Methods. In sum, each simulation produced a new dataset with new estimates of *propmale* for each herd and each year. From those simulated values, we also calculated the 20-year mean and the rolling 3-year average of *propmale,* which were used in the final model.

**Table S5.** Example of a simulated dataset, showing three years of simulated data for one mule deer herd. The simulated data (SIM) is produced by adding observation error (Table S4) to each of the covariate estimates.									
**Herd**	**Year**	**PopSize**	**PopSize SIM**	**Male harvest**	**Male harvest SIM**	**M:F**	**M:F SIM**	**J:F**	**J:F SIM**
Baggs	2000	20300	18848	2033	2019	27	26.8	62	62.2
Baggs	2001	18000	18319	1344	1355	21	21.7	47	46.7
Baggs	2002	20500	20315	1738	1733	19	20.2	49	48.3

Model fitting and parameter estimation

For each simulated dataset, we fit the model below (model a1) and extracted the covariate estimates for $\beta_{meanpropmale}$ and $\beta_{3yr\_rel\_propmale}$.

$$\text{logit}(\text{CWD}_t)=\alpha+\beta_{\text{meanpropmale}}(\text{mean\_propmale}_{\text{herd}})+\beta_{\text{3yr\_rel\_propmale}}(\text{3yr\_rel\_propmale}_{\text{herd,t}})+\text{AR1}+\epsilon_{\text{herd}}$$

This was repeated 100 times (e.g., for 100 simulated datasets with observation error).

Explore Sensitivity

First, we compared the estimates of *propmale* (proportion of males harvested) originally estimated by WGFD and used in the original analysis ('original estimates', x-axis), to estimates of *propmale* simulated using observation uncertainty (y-axis), in order to visualize the magnitude of variation (Figure S1).

Next, we compared parameter estimates from models fit on simulated data to the original model presented in the paper. For each of the 100 simulations, we extracted the parameter estimate for $\beta_{3yr\_rel\_propmale}$, or the effect of the relative harvest level for the past 3 years on CWD prevalence. Parameter estimates from simulated datasets were slightly smaller (closer to 0) than the dataset with no uncertainty (Figure S2). Thus, the effect of harvest is slightly attenuated when the dataset includes uncertain (and more variable) estimates of male harvest. However, the effect of harvest was negative for all simulated datasets and in 80% of

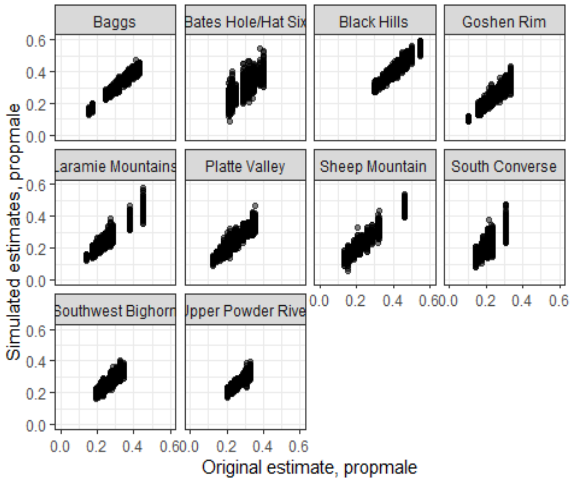

Figure S1. Simulated estimates of proportion of males harvested (propmale), incorporating observation error (y-axis), compared to estimates used in the original analysis (x-axis).

simulated datasets, the effect of harvest was significant (i.e., 95% CI of parameter estimate did not overlap 0).

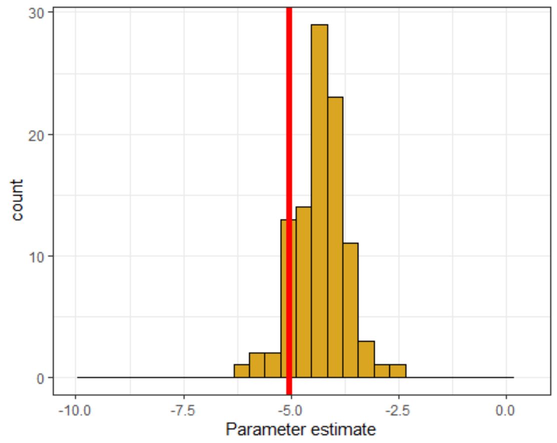

Figure S2. Parameter estimates of the effect of 3-year relative harvest pressure on CWD prevalence $\beta_{3yr\_rel\_propmale}$, for 100 simulated datasets (in gold). The parameter estimate from the original dataset is in red.

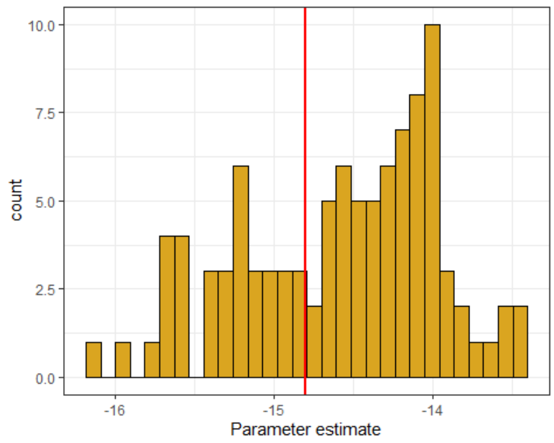

Figure S3. Parameter estimates of the effect of mean harvest pressure on CWD prevalence ($\beta_{mean\_propmale}$), for 100 simulated datasets (in gold). The parameter estimate from the original dataset is in red.

We also examined the how the effect of mean harvest rate ($\beta_{mean\_propmale}$) was affected by covariate uncertainty (Figure S3). This estimate was less affected by uncertainty in the covariates because we averaged across all 20 years of the study, and thus the variation across individual years did not have as strong an influence.

Caveats and Conclusions

Without estimates of error for each covariate in each year, we have made several assumptions. The first is that the magnitude of measurement error does not change systematically with time. Because the methods used to derive harvest estimates and population ratios have not changed substantially, we believe this is a realistic assumption (however, see below). We have also assumed that measurement error is normally distributed, based upon relatively large sample sizes and a relatively normal distribution of covariate estimates.

Our investigation is likely to err on the conservative side for two reasons. First, the measure of uncertainty in population size was derived from the most recent year of the IPM. Because this estimate is not informed by the following years of data, its uncertainty is generally higher than that of previous years. Second, we have drawn errors independently for each covariate, when in reality, error in these estimates is likely to be correlated as a function of factors like herd size and sampling effort.

In sum, our approach was to simulate data to investigate the sensitivity of parameter estimates to measurement error. This revealed that the effect of harvest pressure on CWD prevalence was robust to uncertainty in metrics of harvest pressure. In the future, error in covariates could be more formally integrated into the modeling approach and parameter

estimation, using models that correct for covariate measurement error or a fully Bayesian approach.
